# Journal of Neurodevelopmental Disorders reviewer acknowledgement 2014

**DOI:** 10.1186/s11689-015-9103-z

**Published:** 2015-02-26

**Authors:** Joseph Piven

**Affiliations:** Carolina Institute for Developmental Disabilities, University of North Carolina, Chapel Hill, 100 Renee Lynne Court, Carrboro, NC 27510 USA

## Abstract

We thank all of our reviewers who have contributed to the journal in volume 6 (2014). High quality and timely reviews are critical to the overall quality of the journal. We are committed to providing a unique and important outlet for scholarship regarding neurodevelopmental disorders and are indebted to the outstanding reviewers who have contributed their time over the last year in helping us to reach this goal.

**Brett Abrahams**

United States of America

**Jane Adams**

United States of America

**Maria Arvio**

Finland

**Torsten Baldeweg**

United Kingdom

**Simon Baron-Cohen**

United Kingdom

**Ben A. Barres**

United States of America

**Renata Bartesaghi**

Italy

**Ayelet Ben-Sason**

Israel

**Raphael Bernier**

United States of America

**Nan Bernstein Ratner**

United States of America

**Catalina Betancur**

France

**Dorothy Bishop**

United Kingdom

**Somer Bishop**

United States of America

**Sven Bolte**

Sweden

**Maria Clara Bonaglia**

Italy

**Oliver Braddick**

United Kingdom

**William Brown**

United States of America

**George Capone**

United States of America

**Melissa Carter**

Canada

**Leslie Carver**

United States of America

**Carissa Cascio**

United States of America

**Tony Charman**

United Kingdom

**Abhijit Chaudhuri**

United Kingdom

**John Colombo**

United States of America

**Andrea Contestabile**

Italy

**Gina Conti-Ramsden**

United Kingdom

**Michael Corballis**

New Zealand

**Michael Craig**

United Kingdom

**Steve Dager**

United States of America

**Kim Dalton**

United States of America

**Julie Daniels**

United States of America

**Gabriel Dichter**

United States of America

**Dagmara Dimitriou**

United Kingdom

**Magdalena Dziembowska**

Poland

**Inge-Marie Eigsti**

United States of America

**Christa Einspieler**

Austria

**Peggy Eis**

United States of America

**Jed Elison**

United States of America

**Susan Faja**

United States of America

**Emily Farran**

United Kingdom

**Deborah Fidler**

United States of America

**Susan Folstein**

United States of America

**Tim Fosker**

United Kingdom

**Nathan Fox**

United States of America

**Nouchine Hadjikhani**

United States of America

**Eveline Hagebeuk**

Netherlands

**Brian Hallahan**

Ireland

**Elizabeth Head**

United States of America

**Patricia Howlin**

United Kingdom

**Eric Huang**

United States of America

**Yadong Huang**

United States of America

**Lisa M. Jacola**

United States of America

**Shafali Jeste**

United States of America

**Michael V. Johnston**

United States of America

**Robert Joseph**

United States of America

**Rajesh Kana**

United States of America

**Katherine Karlsgodt**

United States of America

**Walter Kaufmann**

United States of America

**Kelly King**

United States of America

**Bonita Klein-Tasman**

United States of America

**Kami Koldewyn**

United Kingdom

**Nikolaos Kozeis**

Greece

**Silvia Lanfranchi**

Italy

**Hayley Leonard**

United Kingdom

**April Levin**

United States of America

**Ovsanna Leyfer**

United States of America

**Molly Losh**

United States of America

**Eva Loth**

United Kingdom

**Beth Malow**

United States of America

**Christian Marshall**

Canada

**Peter Marshall**

United States of America

**Karla McGregor**

United States of America

**Carolyn Mervis**

United States of America

**Paul Moes**

United States of America

**Anna Victoria Molofsky**

United States of America

**Eric Morrow**

United States of America

**Matt Mosconi**

United States of America

**Kazuhiko Nakabayashi**

Japan

**Masato Nakafuku**

United States of America

**Giovanni Neri**

Italy

**Nora Newcombe**

United States of America

**Craig Newschaffer**

United States of America

**Rowena Ng**

United States of America

**Rob Nicolson**

Canada

**Jose Luis Olmos-Serrano**

United States of America

**Lucy Osborne**

Canada

**Opal Ousley**

United States of America

**Tyler Perrachione**

United States of America

**Isaac Pessah**

United States of America

**Karen Pierce**

United States of America

**Elena Plante**

United States of America

**Daniela Plesa-Skwerer**

United States of America

**Melanie Porter**

Australia

**John R. Pruett Jr**

United States of America

**Zhenghan Qi**

United States of America

**Michael Rafii**

United States of America

**Susan Ravizza**

United States of America

**Keith Rayner**

United States of America

**Francesco Ria**

Italy

**Mabel Rice**

United States of America

**Jane Roberts**

United States of America

**Timothy Roberts**

United States of America

**Randal Ross**

United States of America

**Joanne Rovet**

Canada

**Fabio Sambataro**

Italy

**Carolyn Schanen**

United States of America

**Stephen Scherer**

Canada

**Suzy Scherf**

United States of America

**Michael Schlund**

United States of America

**Sarah Schoen**

United States of America

**Robert Schultz**

United States of America

**Elsa Shapiro**

United States of America

**Frederick Shic**

United States of America

**Tony Simon**

United States of America

**Karun Singh**

Canada

**Liz Smith**

United Kingdom

**Jesse Snedeker**

United States of America

**Mikle South**

United States of America

**Sarah Spence**

United States of America

**Daniel Stahl**

United Kingdom

**Catherine Stamoulis**

United States of America

**Julia Stephen**

United States of America

**Courtney Stevens**

United States of America

**Yujiao Sun**

United States of America

**John Sweeney**

United States of America

**Frank Symons**

United States of America

**Nicole Tartaglia**

United States of America

**Michel Thiebaut de Schotten**

United Kingdom

**Brent Vander Wyk**

United States of America

**Stefano Vicari**

Italy

**Giacomo Vivanti**

Australia

**Jennifer Wagner**

United States of America

**Seth Warschausky**

United States of America

**Sara Webb**

United States of America

**Corrine Welt**

United States of America

**Andrew Whitehouse**

Australia

**Kate Woodcock**

United Kingdom

**Timothy Yu**

United States of America

**Ryan Yuen**

Canada

